# ARRDC3 tyrosine phosphorylation functions as a switch to control c-Src *versus* WWP2 interactions and distinct scaffolding functions

**DOI:** 10.1016/j.jbc.2025.110270

**Published:** 2025-05-21

**Authors:** Mika Caplan, Carolyne Bardeleben, Kanika Dhawan, Rhea Plawat, Irina Kufareva, JoAnn Trejo

**Affiliations:** 1 Department of Pharmacology, School of Medicine, University of California San Diego, La Jolla, California, USA; 2 Biomedical Sciences Graduate Program, University of California San Diego, La Jolla, California, USA; 3 Skaggs School of Pharmacy and Pharmaceutical Sciences, University of California San Diego, La Jolla, California, USA

**Keywords:** ɑ-arrestin, β-arrestin, g protein, GPCR, PAR1, PPxY motif, protease-activated receptor-1, SH2 domain, signaling, trafficking, WW domain

## Abstract

Mammalian α-arrestins are members of the same arrestin family as the ubiquitously expressed and extensively studied **β**-arrestins. Arrestins share common structural elements, including the conserved N- and C-arrestin-fold domains, polar core, finger loop, and C-terminal tail, all of which mediate protein–protein interactions. In **β**-arrestins, these domains enable the control of G protein-coupled receptor (GPCR) signaling and scaffolding interactions with various signaling proteins including c-Src. By contrast, the repertoire of **α**-arrestin scaffolding partners and regulatory mechanisms that control their interactions are not well-understood. **α**-arrestins differ considerably from **β**-arrestins in the C-terminal region; **β**-arrestins contain clathrin adaptor **β**-adaptin-binding sites, whereas **α**-arrestins harbor PPxY motifs, demonstrated to interact with WW domains of E3 ubiquitin ligases such as WWP2. Here we report the identification of a novel phosphorylation site, tyrosine (Y) 394, embedded in the C-terminal PPxY motif of **α**-arrestin ARRDC3. The Y394 site functions as a phospho-regulatory switch to enable distinct ARRDC3 binding partners and scaffolding functions. We found that ARRDC3 Y394 phosphorylation promotes interaction with c-Src *via* its SH2 domain, whereas the non-phosphorylated form binds to WWP2. Our results further show that ARRDC3 Y394 phosphorylation and c-Src SH2 domain-dependent interaction enables regulation of c-Src activity, whereas ARRDC3 Y394 phosphorylation disrupts WWP2 interaction and perturbs ARRDC3-dependent lysosomal trafficking of the GPCR, protease-activated receptor-1. Together, these findings indicate that ARRDC3 Y394 functions as a phospho-regulatory switch to enable selective binding to different partners that impact distinct scaffolding functions.

The mammalian arrestin superfamily includes the visual arrestins, ubiquitously expressed β-arrestins, and more recently identified α-arrestins. The α-arrestins contain the N- and C-arrestin-fold domains, a predicted polar core, and finger loop region (1, 2, 3) like β-arrestins but differ from β-arrestins in the C-terminal region. The β-arrestin C-terminus contains clathrin-binding and clathrin adaptor, β-adaptin-binding sites (4, 5), whereas α-arrestins contain C-terminal PPxY motifs with the exception of ARRDC5, which lacks these sites (6). β-arrestins function primarily to control G protein-coupled receptor (GPCR) desensitization and internalization. In addition, β-arrestins function as remarkable scaffolding proteins to diminish or enhance signaling responses through interactions with various regulatory proteins (1). Of the many proteins that bind to β-arrestins (7), c-Src was the first signaling protein shown to interact with β-arrestins that propagates secondary signaling responses (8, 9). Unlike β-arrestins, the α-arrestins contain PPxY motifs and are known to interact with WW domains of E3 ubiquitin ligases, including WW domain-containing protein-2 (WWP2) (10, 11). The function of β-arrestins is controlled by the activated GPCR conformation and the pattern of C-tail phosphorylation sites that engage the β-arrestin polar core (12, 13). In contrast to β-arrestins, the regulatory mechanisms that control mammalian α-arrestin binding partners and scaffolding functions are not well understood.

The mammalian α-arrestin family comprises six members, including arrestin domain-containing proteins 1 to 5 (ARRDC1-5) and thioredoxin-interacting protein (TXNIP) (6, 14). In the context of GPCR regulation, the α-arrestin ARRDC3 has been studied the most. Previous reports demonstrated that ARRDC3 facilitates recycling of the β2-adrenergic receptor, which sorts through the canonical ubiquitin-ESCRT pathway (15, 16) and plays a critical role in regulating endosomal-lysosomal trafficking of protease-activated receptor-1 (PAR1) and the purinergic P2Y1 receptor that bypasses the canonical ESCRT pathway. The studies showed that both PAR1 and P2Y1R sort to lysosomes *via* ALG-interacting protein X (ALIX), whose ubiquitination is induced by ARRDC3-mediated recruitment of the WWP2 E3 ubiquitin ligase (17, 18, 19). In invasive breast cancer, the loss of ARRDC3 expression results in diminished PAR1 degradation and aberrant signaling (20), whereas ectopic expression of ARRDC3 restores PAR1 endo-lysosomal trafficking and suppresses signaling, invasion, and metastasis (20, 21). In recent work, ARRDC3 was also shown to suppress GPCR-induced Hippo pathway signaling independently of its effects on receptor trafficking (21), indicating that ARRDC3 is a multifunctional scaffolding protein like β-arrestins. Thus, ARRDC3 exhibits multidimensional functions, including regulation of receptor trafficking and, separately, control of GPCR signaling.

To understand the multiplicity of ARRDC3 functions, a proteomic analysis of the ARRDC3 interactome was conducted and revealed numerous interacting partners including c-Src (22). However, the regulatory mechanisms that control ARRDC3-c-Src interaction and its functional implications are unknown and were examined. Here, we report the identification of a new phosphorylated tyrosine residue in the C-terminal PPxY motif that functions as a phospho-regulatory switch that enables ARRDC3 binding to c-Src *versus* WWP2. Our studies further demonstrate that the ARRDC3 tyrosine phosphorylation-mediated c-Src interaction regulates c-Src activity, whereas tyrosine phosphorylation of ARRDC3 disrupts WWP2 interaction and perturbs lysosomal trafficking of PAR1.

## Results

### c-Src interacts with ARRDC3 independent of **β**-arrestins

**Figure 1:**
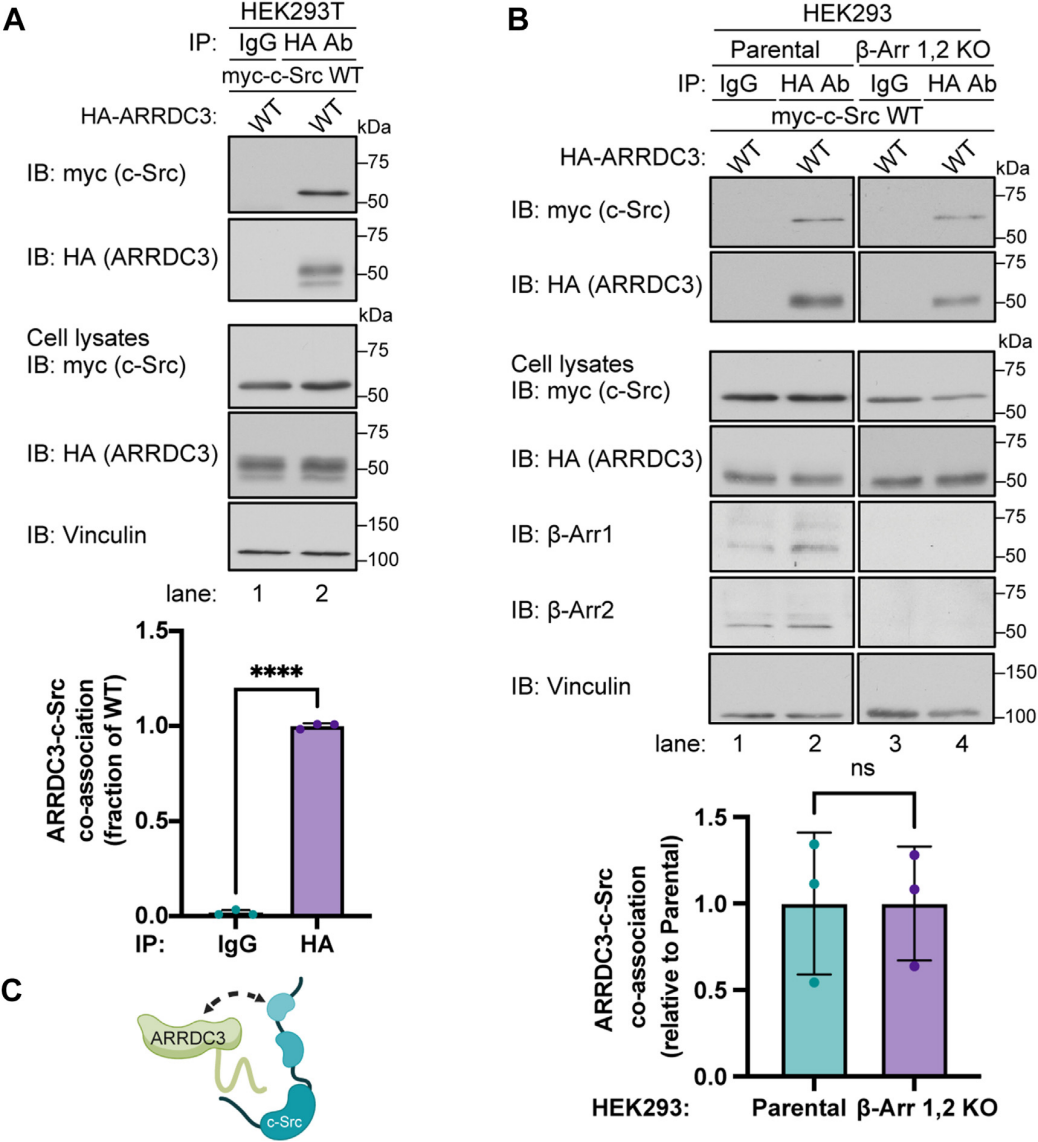
**ARRDC3 interacts with c-Src independent of β-arrestins**. *A*, HEK293T cells expressing HA-ARRDC3 wild-type (WT) and myc-Src WT were immunoprecipitated with anti-HA or IgG antibody, processed and immunoblotted for HA (ARRDC3) and myc (c-Src). ARRDC3-c-Src co-association was quantified (mean ± S.D.) from three independent experiments and analyzed by Student’s *t* test, ∗∗∗∗, *p* < 0.0001. *B*, HEK293 parental and β-arrestin 1,2 CRISPR/Cas9 knockout (KO cells) co-expressing HA-ARRDC3 and myc-Src were immunoprecipitated and co-association quantified from three independent experiments. Data (mean ± S.D.) were analyzed by Student’s *t* test, ns = not significant. *C*, Cartoon of ARRDC3-c-Src interaction.

To assess α-arrestin ARRDC3 interaction with c-Src, HA-ARRDC3 and myc-c-Src were transiently expressed in HEK293T cells, and association was examined by co-immunoprecipitation (co-IP). In cells co-expressing myc-c-Src and HA-ARRDC3, c-Src was detected in immunoprecipitates of HA-ARRDC3 and not in IgG control IPs (Fig. 1*A*, lane 1 vs. 2). β-arrestins can similarly interact with c-Src (8, 9) and previous studies showed that ARRDC3 can dimerize with β-arrestins (23). Thus, to determine whether c-Src-ARRDC3 association is mediated by β-arrestins, we utilized HEK293 CRISPR/Cas9 β-arrestin-1, 2 knockout cells (24). In β-arrestin knockout cells, ARRDC3-c-Src co-association was assessed by co-IPs and remained intact, like that observed in parental HEK293 cells (Fig. 1*B* lanes 3–4 vs. 1–2). These findings suggest that ARRDC3-c-Src interaction is independent of β-arrestins (Fig. 1*C*).

### ARRDC3 co-associates with endogenous c-Src and WWP2

**Figure 2:**
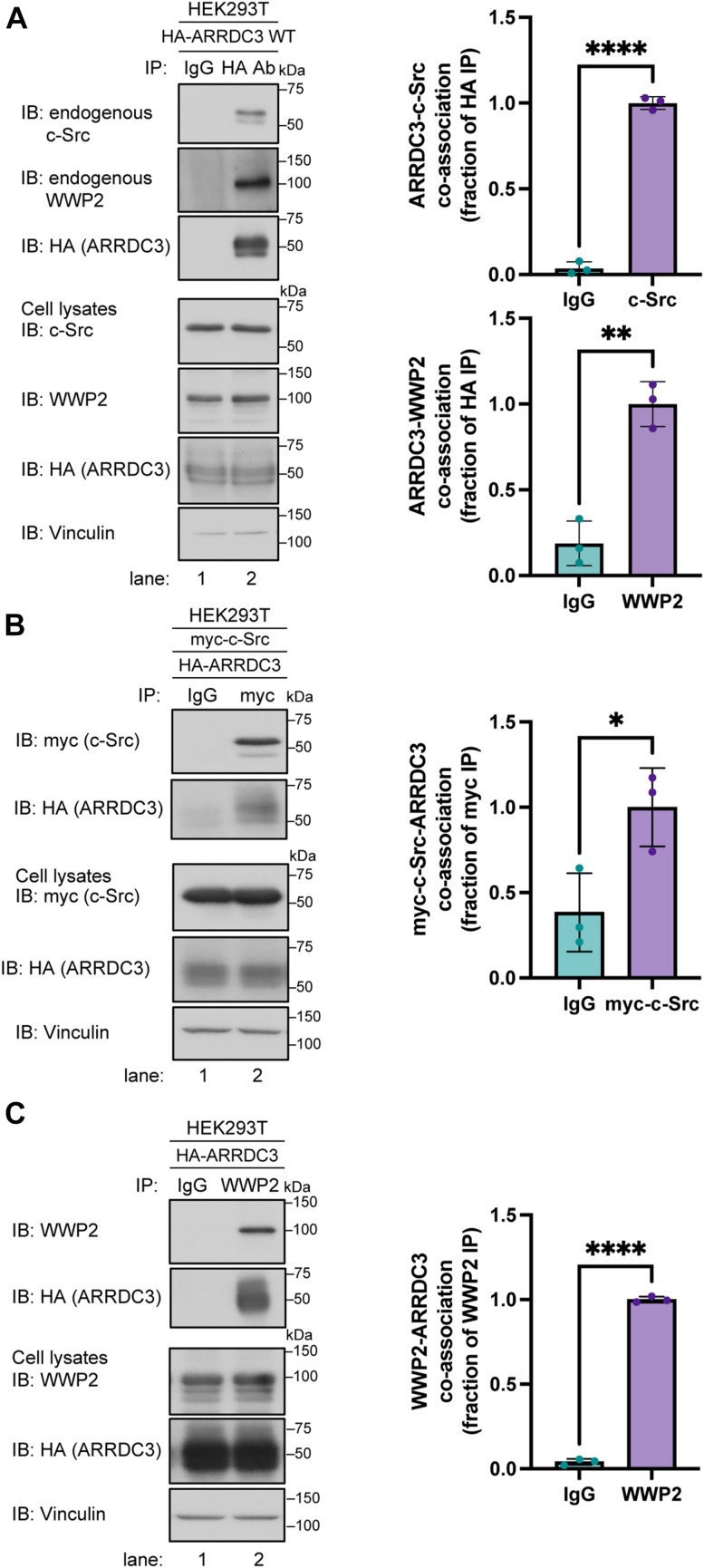
**ARRDC3 interacts with endogenous c-Src and WWP2**. *A*, HEK293T cells expressing HA-ARRDC3 were immunoprecipitated with anti-HA or IgG antibody, processed, and immunoblotted for HA (ARRDC3) and endogenous c-Src and WWP2. ARRDC3-c-Src and ARRDC3-WWP2 co-association was quantified (mean ± S.D.) from three independent experiments and analyzed by Student’s *t* test, ∗∗∗∗, *p* < 0.0001, ∗∗, *p* = 0.0016. *B*, HEK293T cells expressing HA-ARRDC3 and myc-c-Src were immunoprecipitated with anti-myc or IgG antibody, processed, and immunoblotted for myc (c-Src) and HA (ARRDC3). Myc-c-Src-ARRDC3 co-association was quantified (mean ± S.D.) from three independent experiments and analyzed by Student’s *t* test, ∗, *p* = 0.03. *C*, HEK293T cells expressing HA-ARRDC3 were immunoprecipitated with anti-WWP2 or IgG antibody, processed, and immunoblotted for HA (ARRDC3) and WWP2. WWP2-ARRDC3 co-association was quantified (mean ± S.D.) from three independent experiments and analyzed by Student’s *t* test, ∗∗∗∗, *p* < 0.0001.

Next, we examined the capacity of HA-ARRDC3 to interact with endogenous c-Src and WWP2 in the same cell population. HA-ARRDC3 expressed in HEK293T cells was immunoprecipitated, and both endogenous c-Src and WWP2 were detected in the IP (Fig. 2*A*, lanes 1 *versus* 2). These findings indicate that endogenous c-Src and WWP2 can co-associate with ARRDC3 in the same cells. To confirm ARRDC3 interaction with c-Src and WWP2, we performed reciprocal immunoprecipitations using anti-myc antibodies or WWP2 antibodies. In the myc-c-Src IP from cells expressing myc-c-Src and HA-ARRDC3, a robust co-association of myc-c-Src with ARRDC3 was observed (Fig. 2*B*, lanes 1 *versus* 2). Similarly, in the IP of WWP2, a substantial interaction of WWP2 with HA-ARRDC3 was detected (Fig. 2*C*, lanes 1 *versus* 2). Taken together, these results suggest that ARRDC3 interacts with both c-Src and WWP2.

### c-Src SH2 phospho-tyrosine binding domain is required for interaction with ARRDC3

**Figure 3:**
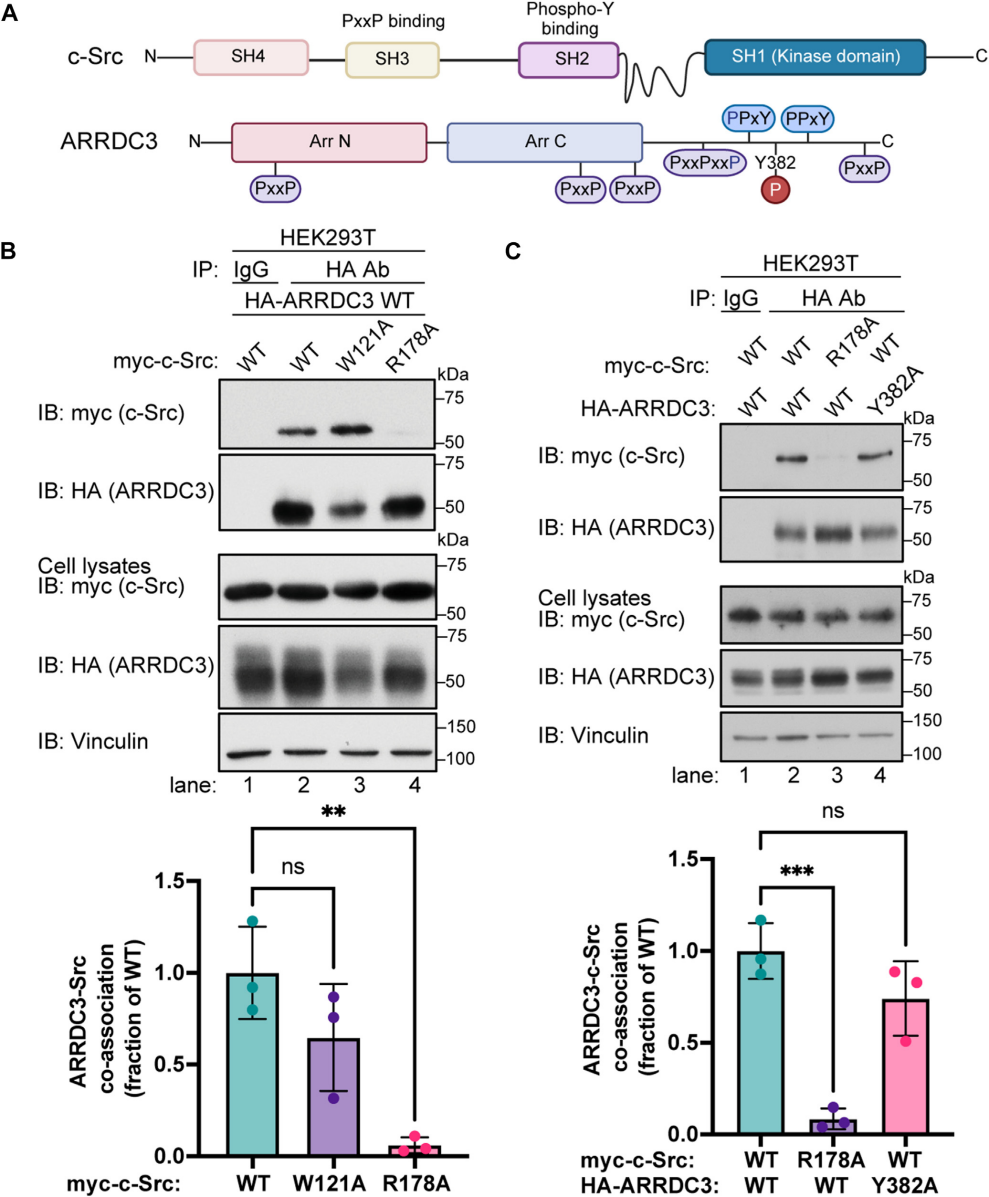
**c-Src SH2 phospho-tyrosine binding domain is required for interaction with ARRDC3**. *A*, schematic of c-Src and ARRDC3 protein domains. c-Src contains an SH1 kinase domain, an SH2 phospho-tyrosine recognition domain, an SH3 PxxP binding domain, and a unique SH4 domain. ARRDC3 contains N- and C-arrestin-fold domains and five PxxP motifs and a phosphorylated tyrosine (Y)382 residue. *B*, HEK293T cells expressing HA-ARRDC3 wild-type (WT) and either c-Src WT, SH3 domain W121A mutant, or SH2 domain R178A mutant were immunoprecipitated and immunoblotted as indicated. ARRDC3-c-Src co-association was quantified (mean ± S.D.) from 3 independent experiments and analyzed by one-way ANOVA followed by Dunnett's multiple comparisons test; ∗∗, *p* < 0.006, ns = not significant. *C*, HEK293T cells expressing myc-Src WT or R187A mutant and either HA-ARRDC3 WT or Y382A mutant were immunoprecipitated and immunoblotted as indicated. Data (mean ± S.D.) were quantified from three independent experiments and analyzed by one-way ANOVA followed by Dunnett’s multiple comparisons test, ∗∗∗, *p* = 0.0008, ns = not significant.

C-Src contains a Src homology 3 (SH3) domain that recognizes short linear motifs with a PxxP signature, a Src homology 2 (SH2) domain that binds phospho-tyrosine (Y) motifs, and a catalytic Src homology 1 (SH1) kinase domain (Fig. 3*A*). ARRDC3 is composed of arrestin-like N- and C-domains and a C-terminal tail (C-tail), which contains five proline (P) rich PxxP motifs, two PPxY motifs and a Y382 residue previously reported to be phosphorylated that is not part of the PPxY motifs (Fig. 3*A*) (25, 26). To define the c-Src domains that mediate interaction with ARRDC3, we examined HA-ARRDC3 co-association with a myc-c-Src SH2 arginine (R) 178 to alanine (A) mutant, which disrupts the essential phospho-tyrosine recognition residue (27) and SH3 tryptophan (W) 121A mutant in HEK293T cells, which is essential for SH3 domain binding to PxxP motifs (28, 29). ARRDC3 robustly co-immunoprecipitated with wild-type c-Src and the c-Src SH3 W121A mutant but failed to interact with the SH2 R178A mutant of c-Src (Fig. 3*B*, lanes 2–4). Thus, c-Src SH2 phospho-tyrosine binding domain likely mediates the association with ARRDC3, suggesting that the interaction may require a phosphorylated tyrosine residue on ARRDC3.

To probe the function of the previously identified ARRDC3 Y382 phosphosite, HEK293T cells were transiently transfected with either HA-ARRDC3 wild-type or Y382A mutant together with myc-c-Src wild-type and SH2 R178A mutant, and interaction was determined by co-IP. Remarkably, the ARRDC3 Y382A mutant retained the capacity to bind to c-Src like wild-type ARRDC3 (Fig. 3*C*, lanes 1–2 vs. 4), and as expected, the c-Src SH2 R178A mutant failed to bind to wild-type ARRDC3 (Fig. 3*C*, lanes 1–3). Although phosphorylation of the Y382 site has been reported (25, 26), these results suggest that ARRDC3-c-Src interaction is mediated by a phosphotyrosine different from Y382.

### A newly identified ARRDC3 Y394 phospho-site mediates interaction with c-Src

**Figure 4:**
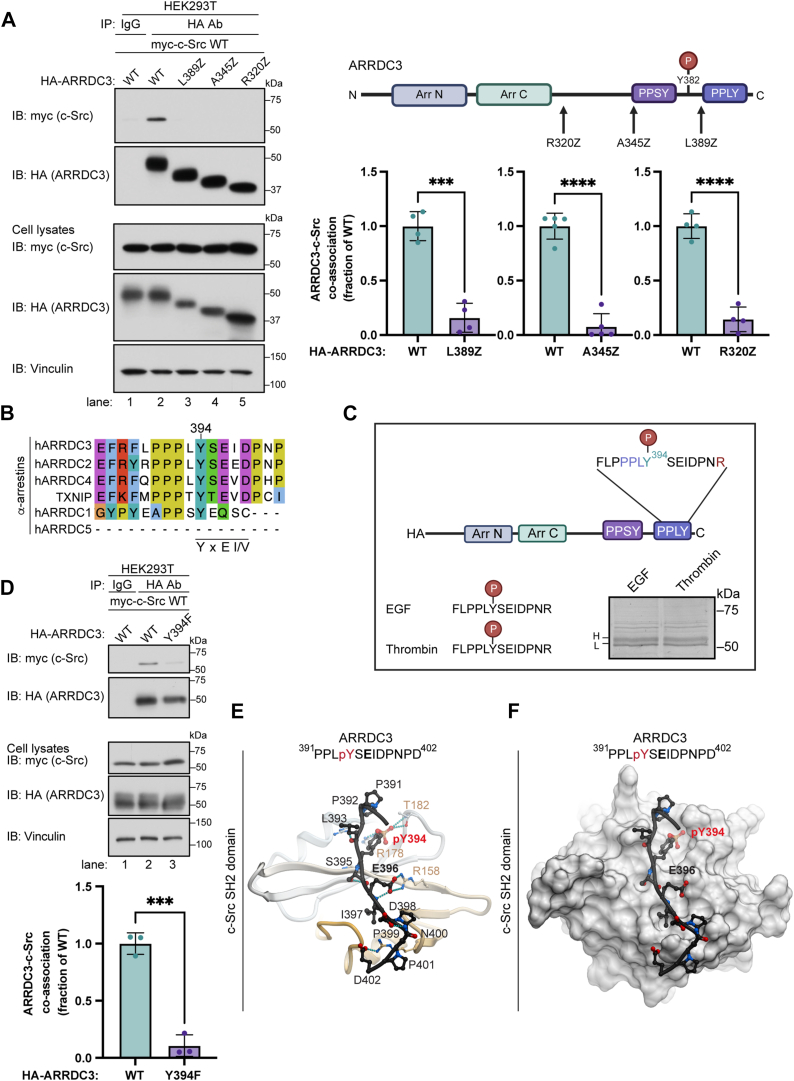
**A newly identified ARRDC3 Y394 phospho-site mediates interaction with c-Src**. *A*, HEK293T cells co-expressing myc-Src and either HA-ARRDC3 wild-type (WT) or ARRDC3 L389Z, A345Z, and R320Z C-tail truncation mutants were immunoprecipitated and immunoblotted as indicated. Schematic of ARRDC3 indicates the point (*arrow*) of truncation. ARRDC3-c-Src co-association was quantified (mean ± S.D.) from four independent experiments and analyzed by Student’s *t* test; ∗∗∗, *p* = 0.001; ∗∗∗∗, *p* < 0.0001. *B*, alignment of human ɑ-arrestin C-tail PPxY motif, Y394 and YxEI/V is the semi-conserved c-Src SH2 consensus binding site. *C*, HEK293T cells expressing HA-ARRDC3 P401R variant were stimulated with EGF (50 ng/ml) or thrombin (10 nM) for 5 min, processed and both upper and lower bands were subjected to mass spectrometry. ARRDC3 Y394 phosphorylation was detected in same peptide in EGF and thrombin-stimulated cells. *D*, HEK293T cells expressing myc-c-Src and either HA-ARRDC3 wild-type (WT) or Y394F mutant, immunoprecipitated and immunoblotted as indicated. ARRDC3-c-Src co-association was quantified (mean ± S.D.) from three independent experiments and analyzed by Student’s *t* test; ∗∗∗, *p* < 0.0003. *E*–*F*, a molecular model of the c-Src SH2 domain comprised of amino acids (aa) 144 to 249 with the C-terminal peptide (aa 391–402) from ARRDC3 C-tail. c-Src SH2 is shown as a ribbon in (*E*) and as a space-filling model in (*F*). The ARRDC3 peptide is shown as a *black ribbon* and *sticks*, whereas the phosphorylated Y394 residue is shown in *red*. In (*E*), polar interactions are emphasized, and hydrogen bonds are shown as *cyan dotted lines*. In (*F*), the c-Src SH2 domain surface mesh emphasizes the cavity that accommodates ARRDC3 pY394.

To map the ARRDC3 region that mediates interaction with c-Src, a series of HA-ARRDC3 C-tail truncation mutants lacking either 94 amino acids (aa) in R320 termination codon (Z), 69 aa in A345Z or 25 aa in leucine (L) 389Z were tested for their capacity to interact with myc-c-Src in HEK293T cells (Fig. 4*A*). While full-length ARRDC3 specifically interacted with c-Src (Fig. 4*A*, lanes 1–2), all the ARRDC3 C-tail deletion mutants failed to co-associate with c-Src (Fig. 4*A*, lanes 3–5). These results indicate that the distal C-tail region (aa 389–414) may be important. Unexpectedly, this region contains only a single tyrosine, Y394, which is part of the second PPxY motif of ARRDC3.

C-Src SH2 domains bind with high affinity to substrate pYEEI consensus sites, where the key Y residue is phosphorylated (30). Sequence alignment of the six mammalian α-arrestins C-tail PPxY region encompassing Y394 revealed a semi-conserved YSEI motif in ARRDC3, as well as a YS/TEV motif in ARRDC4 and TXNIP (Fig. 4*B*). Using ScanSite 4.0 prediction analysis software (31), the ARRDC3 Y394 residue was predicted to bind to the Src family kinase Fyn SH2 domain (Table S1 and Fig. S1). Next, we used mass spectrometry to determine the phosphorylation status of the ARRDC3 Y394 site. HEK293T cells expressing HA-ARRDC3 were stimulated for 5 min with thrombin to activate PAR1 or epidermal growth factor (EGF) to activate EGFR, a receptor tyrosine kinase. After agonist stimulation, purified HA-ARRDC3 was resolved by SDS-PAGE gel and found to migrate as a lower and higher molecular weight band. The bands were excised separately and independently subjected to trypsin digest and analyzed by mass spectrometry (Fig. 4*C*). Y394 phosphorylation was detected in ARRDC3 phospho-peptides in cells stimulated with thrombin and cells stimulated with EGF (Fig. 4*C* and Fig. S2). To determine whether ARRDC3 Y394 phosphorylation is required for interaction with c-Src, a non-phosphorylatable ARRDC3 Y394F mutant was generated by replacing Y with phenylalanine (F). While wild-type ARRDC3 showed robust interaction with c-Src, the ARRDC3 Y394F mutant co-association with c-Src was nearly abolished (Fig. 4*D*, lanes 1–3). Thus, ARRDC3 Y394 phosphorylation is likely the key determinant for binding to the SH2 domain of c-Src.

Next, we used homology modeling to explore interactions between the ARRDC3 C-tail region (as 391–402), including the Y394 phospho-site and the SH2 domain of c-Src. The modeling predicted a direct interaction between the phospho-Y394 residue and the conserved loop formed by the c-Src SH2 domain (Fig. 4, *E* and *F*). The modeling also showed that the ARRDC3 glutamic acid (E396), conserved in most mammalian ɑ-arrestins (Fig. 4*B*), plays a critical role in stabilizing the interaction with hydrogen bonds to R158 in the SH2 domain of c-Src. Together, these findings indicate that the ARRDC3 Y394 phospho-site can indeed mediate interaction with the SH2 domain of c-Src.

### Y394 phosphorylation regulates binding of ARRDC3 to c-Src *versus* WWP2

**Figure 5:**
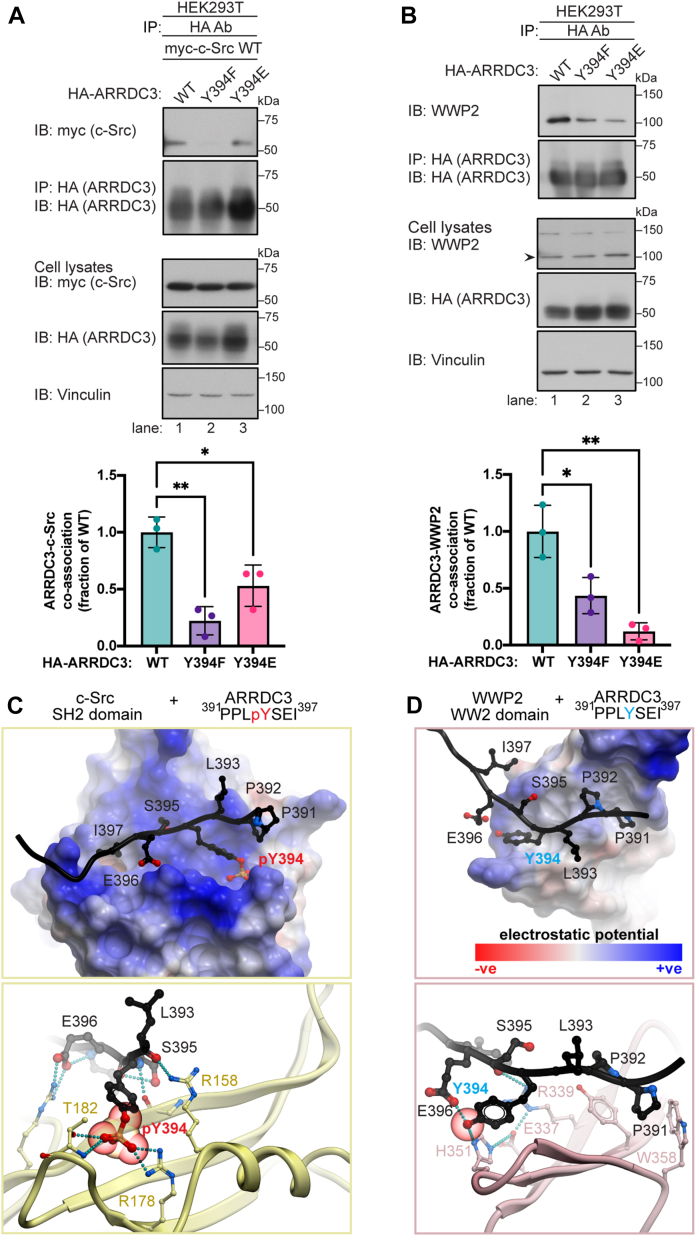
**Y394 phosphorylation controls binding of ARRDC3 to c-Src *versus* WWP2**. *A*, HEK293T cells expressing myc-c-Src wild-type (WT) and HA-ARRDC3 WT, Y394F, or Y394E mutant were lysed, immunoprecipitated with HA magnetic beads, and immunoblotted as indicated. Data (mean ± S.D.) from three independent experiments were quantified and analyzed by one-way ANOVA followed by Tukey’s *post hoc* test; ∗∗, *p* < 0.0017, ∗, *p* < 0.0194. *B*, HEK293T cells expressing HA-ARRDC3 WT, Y394F, or Y394E mutant were lysed, immunoprecipitated, and endogenous WWP2 (*arrowhead*) detected by immunoblotting. Data (mean ± S.D.) were quantified from three independent experiments and analyzed by one-way ANOVA followed by Tukey’s *post hoc* test; ∗, *p* < 0.0144, ∗∗, *p* < 0.0016. *C*, a 3D model of a complex between the C-terminal peptide of ARRDC3 (residues 390–402, *black ribbon* and *sticks*), including a phosphorylated Y394 residue, and the SH2 domain of c-Src (residues 144–249). In the *top panel*, c-Src SH2 domain is shown as a surface mesh and colored by electrostatic potential; in the *bottom panel*, c-Src SH2 domain is shown in *yellow ribbon* and *sticks* and a close-up view of pY394 and its interacting residues is shown. *D*, a 3D model of a complex between the C-terminal peptide of ARRDC3 (residues 390–402) and the WW2 domain of WWP2 (residues 296–369). In the *top panel*, WWP2 WW2 domain is shown as a surface mesh and colored by electrostatic potential; in the *bottom panel*, WWP2 WW2 domain is shown in *pink ribbon* and *sticks* and a close-up view of non-phosphorylated Y394 and its interacting residues is shown.

While we show that ARRDC3 Y394 phosphorylation mediates binding to the c-Src SH2 domain, Y394 is part of the second PPxY motif and critical for binding to WW domains of the E3 ubiquitin ligase WWP2 (11). We hypothesize that Y394 phosphorylation may act as a switch to facilitate ARRDC3 binding to distinct partners. To test this hypothesis, we examined the capacity of an ARRDC3 phospho-mimetic Y394 to glutamic acid (E) *versus* a non-phosphorylatable Y394F mutant to interact with c-Src and WWP2. HEK293T cells were transfected with myc-c-Src wild-type and either HA-ARRDC3 wild-type, Y394F, or Y394E mutant, and the co-association between myc-c-Src and HA-ARRDC3 variants was examined by co-IP. C-Src association with ARRDC3 Y394F mutant was nearly abolished, resulting in a significant reduction of binding compared to wild-type ARRDC3 (Fig. 5*A*, lanes 1–2). c-Src retained a greater capacity to bind to ARRDC3 Y394E phospho-mimetic *versus* non-phosphorylatable Y394F mutant (Fig. 5*A*, lanes 2–3), although binding was partially reduced compared to ARRDC3 wild-type (Fig. 5*A*, lanes 1 vs. 3). These results are consistent with c-Src binding to the phosphorylated Y394 site of ARRDC3. Next, we examined whether Y394 phosphorylation is important for ARRDC3-WWP2 interaction. In contrast to c-Src, WWP2 interaction with the Y394E phospho-mimetic was significantly reduced, resulting in almost complete loss of co-association compared to wild-type ARRDC3 (Fig. 5*B*, lanes 1 vs. 3). However, WWP2 showed a greater capacity to bind to the Y394F non-phosphorylatable mutant (Fig. 5*B*, lanes 1–2) compared to c-Src, which displayed a substantial loss in binding to ARRDC3 Y394 F (Fig. 5*A*, lanes 1–2). These data support the notion that ARRDC3 Y394 phosphorylation regulates binding to c-Src *versus* WWP2.

Molecular modeling was next used to understand the structural basis for ARRDC3 preferences. The model of c-Src with a seven amino acid, pY394-containing ARRDC3 peptide suggests that the peptide binds in a positively charged groove on the SH2 domain (Fig. 5*C*) and forms numerous charge-charge and hydrogen bonding contacts. The pY394 phosphate group alone makes as many as 5 hydrogen bonds with conserved R178 and T182 residues of c-Src, whereas the nearby ARRDC3 S395 and E396 create additional polar interactions with c-Src R158 and R208 (Fig. 5*C*, lower box). In contrast, the model of WWP2 with the unphosphorylated Y394 form of the same peptide demonstrates that its binding groove on the WW2 domain is neutral in charge (Fig. 5*D*), and the interaction is driven by the packing of P391 and P392 of ARRDC3 PPxY motif against WWP2 W358 and assisted by hydrogen bonds that the uncharged hydroxyl of ARRDC3 Y394 makes with ARRDC3 E396 and WWP2 H351 (Fig. 5*D* lower box). These favorable interactions support the idea that c-Src prefers the phosphorylated Y394 of ARRDC3, whereas WWP2 binds stronger to the unphosphorylated Y394 form. The models also demonstrate that the phospho-mimetic Y394E and non-phosphorylatable Y394F mutants are rather poor mimics of the corresponding phosphorylated Y394 or unphosphorylated Y394 forms of ARRDC3. For example, unlike phospho-Y394 that can form up to 7 hydrogen bonds, in a tetrahedral geometry, E394 can only form 4 and is planar/sp2-hybridized. Similarly, unlike the Y394 whose hydroxyl can form two hydrogen bonds, F394 is unable to do so. This explains why both ARRDC3 mutations led to a reduction in binding to both c-Src and WWP2, compared to wild-type ARRDC3, albeit to a different extent. Taken together, these findings suggest that the ARRDC3 Y394 functions as a switch to control selective binding of two distinct partners, c-Src and WWP2.

### ARRDC3 unphosphorylatable Y394F mutant alters thrombin-stimulated c-Src activity

**Figure 6:**
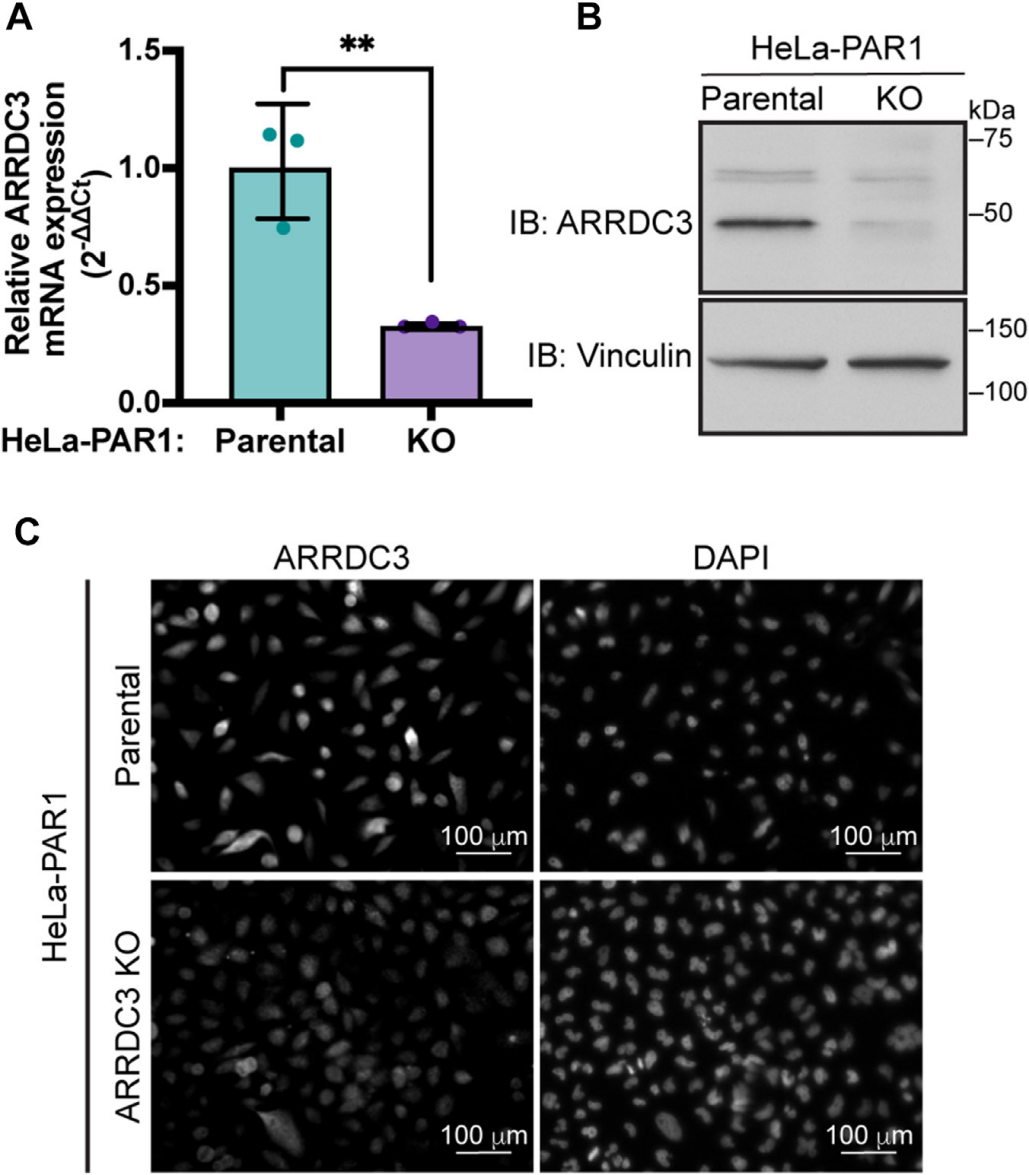
**Characterization of HeLa-PAR1 ARRDC3 CRISPR/Cas9 knockout cell line**. *A*, ARRDC3 mRNA transcript abundance was quantified in HeLa-PAR1 parental cells and HeLa-PAR1 ARRDC3 CRISPR/Cas9 knockout (KO) cells (mean ± S.D., n = 3) and analyzed by Student’s *t* test; ∗∗, *p* < 0.0063. *B*, HeLa-PAR1 parental and ARRDC3 KO cells were lysed and ARRDC3 protein expressed expression measured by immunoblot. *C*, HeLa-PAR1 parental and ARRDC3 KO cells were processed, immunostained for ARRDC3 and imaged by confocal microscopy. DAPI was used to detect nuclei. Scale bar, 100 μm.

ARRDC3 is a multifunctional adaptor protein known to control GPCR lysosomal trafficking and is also able to suppress GPCR signaling independent of receptor trafficking (19, 21). However, the implications of Y394 phosphorylation in ARRDC3 multifaceted functions are not known and were examined. To examine wild-type and mutant ARRDC3 function, we generated an ARRDC3 gene knockout (KO) using CRISPR/Cas9 gene editing in HeLa cells stably expressing FLAG-tagged PAR1. HeLa-PAR1 ARRDC3 KO cells showed significantly lower ARRDC3 mRNA transcript and protein expression compared to parental HeLa-PAR1 (Fig. 6, *A* and *B*). A substantial loss of endogenous ARRDC3 expression was similarly demonstrated in the HeLa-PAR1 ARRDC3 KO cells *versus* parental wild-type HeLa-PAR1 cells using immunofluorescence microscopy (Fig. 6*C*).

**Figure 7:**
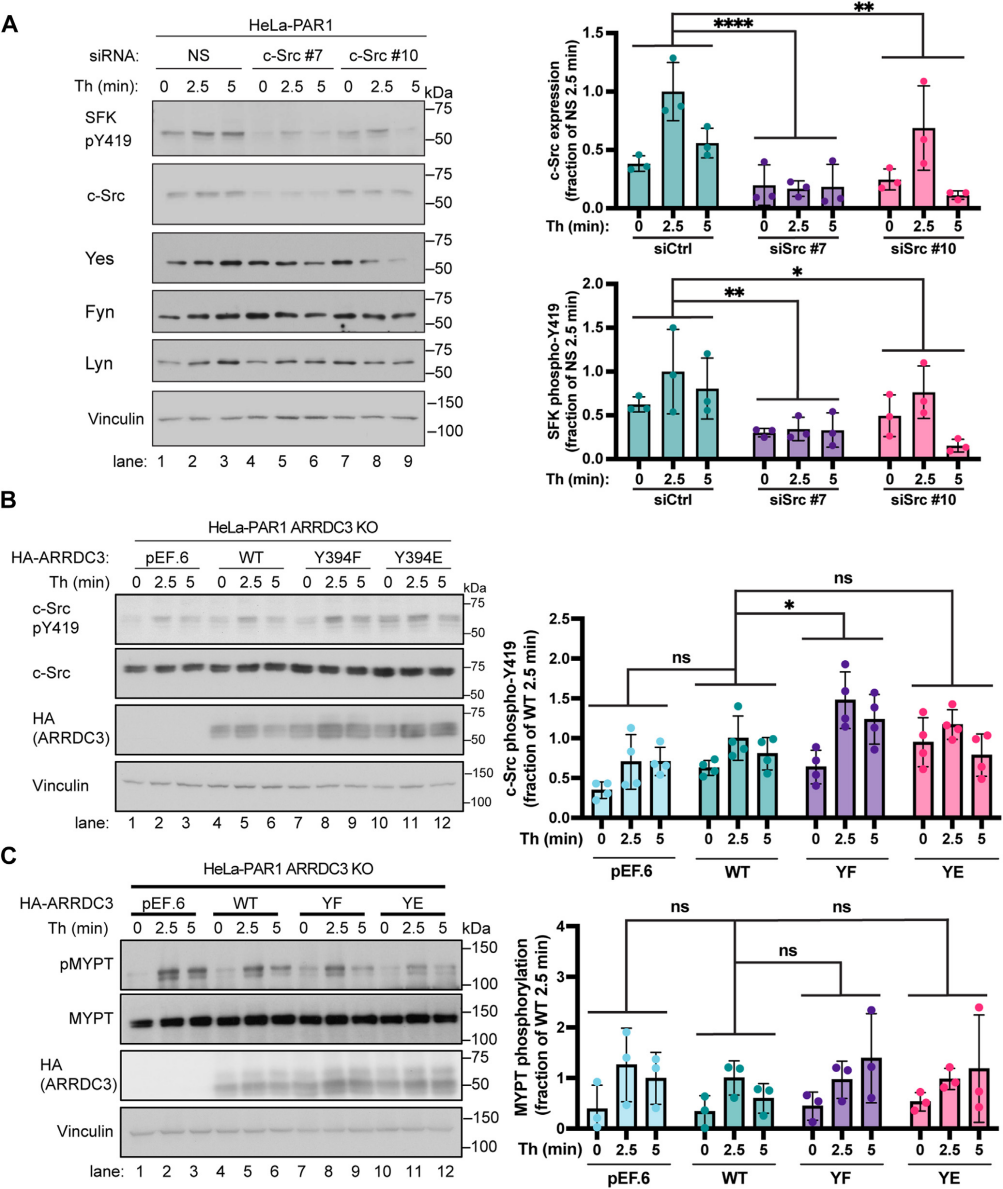
**ARRDC3 unphosphorylatable Y394F mutant alters thrombin-stimulated c-Src activity**. *A*, HeLa-PAR1 transfected with NS or two different c-Src siRNA were stimulated with 10 nM thrombin for various times, lysed and immunoblotted as indicated. Data (mean ± S.D.) were quantified from three independent experiments. C-Src expression analyzed by two-way ANOVA followed by Dunnett’s *post hoc* test; ∗∗∗∗, *p* < 0.0001, ∗∗, *p* = 0.0049 and SFK phosphorylation was examined by two-way ANOVA followed by Dunnett’s *post hoc* test; ∗∗, *p* = 0.0013, ∗, *p* = 0.0195. *B* and *C*, HeLa-PAR1 ARRDC3 KO cells expressing control vector pEF.6, HA-ARRDC3 wild-type (WT), Y394F, or Y394E mutant were stimulated with thrombin, lysed and immunoblotted as indicated. Data (mean ± S.D.) from four independent experiments are shown as changes in c-Src Y419 phosphorylation normalized to total c-Src and analyzed by two-way ANOVA followed by Dunnett’s *post hoc* test; WT vs. YF, ∗, *p* = 0.0239, ns = not significant. The data (mean ± S.D.) from three independent experiments for MYPT phosphorylation were normalized to total MYPT and analyzed by two-way ANOVA followed by Dunnett’s *post hoc* test; ns = not significant.

A key event in c-Src activation is the induction of conformational changes that enables release from the autoinhibited state, resulting in trans-autophosphorylation at Y419, which stabilizes the activation loop (32). Thus, to examine the effect of ARRDC3 wild-type and mutants on thrombin-stimulated c-Src activity, we measured c-Src Y419 phosphorylation utilizing a Y419 phospho-specific antibody. Since activation of Src family kinase (SFK) members *via* Y419 phosphorylation is highly conserved, c-Src-specific siRNAs were first used to verify that thrombin-induced Y419 phosphorylation was attributable to c-Src in HeLa cells. In parental HeLa-PAR1 cells, c-Src protein expression was markedly decreased by c-Src siRNAs compared to cells transfected with non-specific siRNA (Fig. 7*A*, lanes 4–9 vs. 1–3), whereas expression of the SFKs Yes, Fyn, and Lyn remained intact in all siRNA-transfected conditions (Fig. 7*A*, lanes 1–9). These data indicate that the siRNAs specifically deplete c-Src expression without altering the expression of other SFKs. In cells transfected with non-specific siRNA, thrombin induced a marked increase in Y419 phosphorylation at 2.5 min that was sustained for 5 min (Fig. 7*A*, lanes 1–3). By contrast, in cells depleted of c-Src by siRNA, a significant loss of thrombin-induced c-Src Y419 phosphorylation was observed compared to non-specific siRNA-transfected cells (Fig. 7*A*, lanes 4–9 vs. lanes 1–2). Thus, thrombin-stimulated SFK Y419 phosphorylation is dependent on c-Src expression in HeLa cells.

To determine the effect of ARRDC3 Y394 phosphorylation on thrombin-induced c-Src activity, HeLa-PAR1 ARRDC3 KO cells transfected with HA-ARRDC3 wild-type, Y394F non-phosphorylatable or Y394E phospho-mimetic mutants were stimulated with thrombin and c-Src Y419 phosphorylation was detected. Recall, the ARRDC3 Y394F mutant exhibits diminished c-Src binding, whereas the Y394E mutant retains c-Src binding. In cells expressing the ARRDC3 Y394F mutant, thrombin induced a significantly greater increase in c-Src Y419 phosphorylation compared to cells expressing wild-type ARRDC3 or vector control (Fig. 7*B*, lanes 7–9 vs. 1–6), suggesting that ARRDC3-c-Src interaction suppresses c-Src activity. In contrast, c-Src Y419 phosphorylation induced by thrombin in cells expressing the Y394E mutant was comparable to wild-type ARRDC3 (Fig. 7*B*, lanes 10–12 vs. 4–6). To test if the effect of ARRDC3 on PAR1 c-Src signaling is specific, we examined thrombin-induced phosphorylation of myosin phosphatase-targeting protein-1 (MYPT), a downstream effector of G protein-mediated RhoA signaling. Thrombin stimulated a significant increase in MYPT phosphorylation in cells expressing ARRDC3 WT that was comparable in cells expressing Y394F and Y394E mutants and vector control (Fig. 7*C*, lanes, 1–9), suggesting that ARRDC3 does not globally impact thrombin-activated PAR1 signaling. Thus, disruption of the ARRDC3-c-Src interaction due to a non-phosphorylatable Y394F mutation specifically increases thrombin-activated PAR1-induced c-Src activity, suggesting that the binding of ARRDC3 to c-Src suppresses its activity.

### ARRDC3 phospho-mimetic Y394E mutant decreases PAR1 lysosomal trafficking

**Figure 8:**
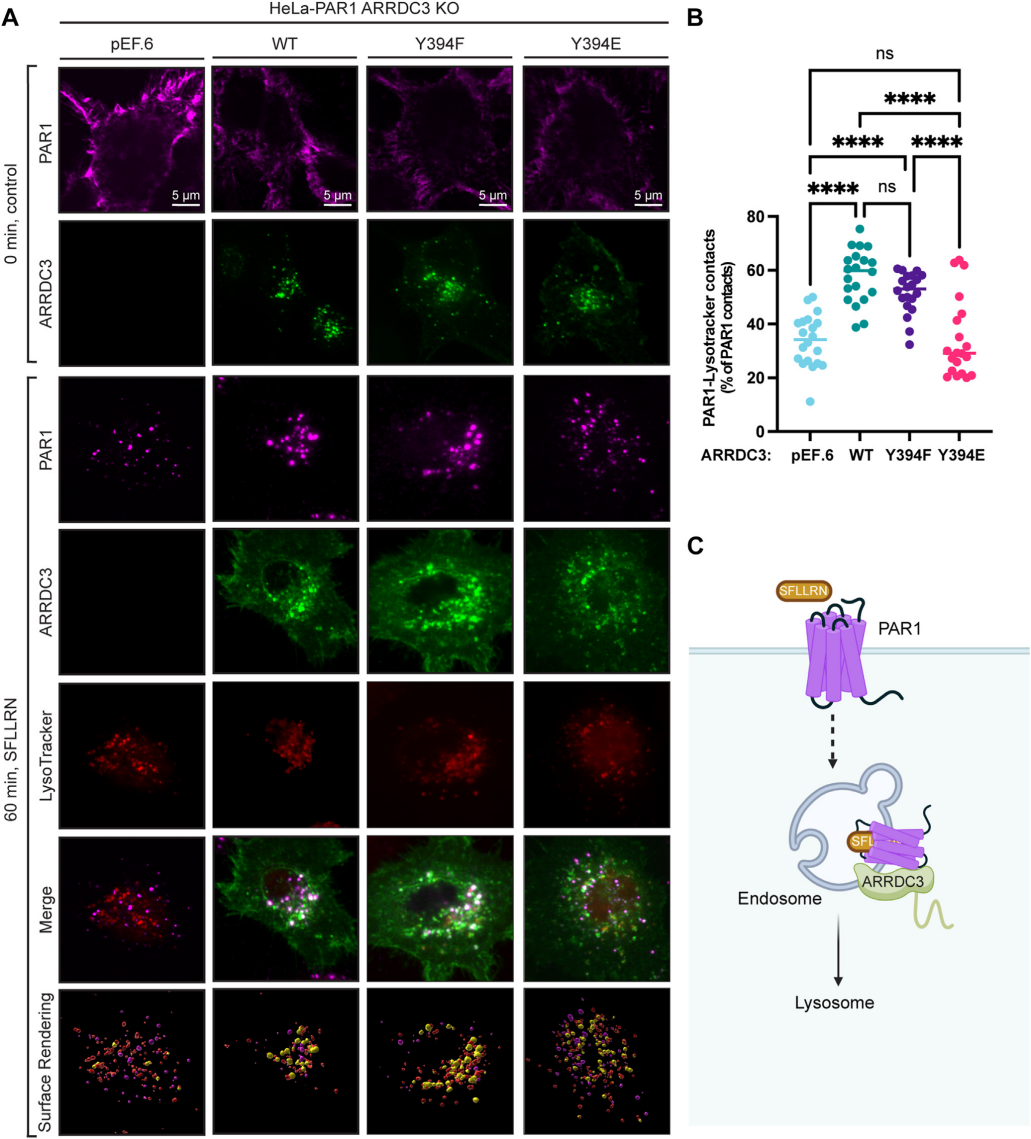
**ARRDC3 Y394 phospho-mimetic decreases PAR1 lysosomal trafficking**. *A*, HeLa-PAR1 ARRDC3 CRISPR/Cas9 knockout (KO) cells expressing HA-ARRDC3 wild-type (WT), Y394F, Y394E, or pEF.6 vector control were stimulated with or without agonist peptide SFLLRN (100 μM), processed and immunostained for HA-ARRDC3 (*green*), PAR1 (*magenta*). LysoTracker (*red*) was used to detect lysosomes. Cells were imaged by confocal microscopy and colocalization examined in the merge images and surface rendering using Imaris software. PAR1-lysosomal colocalization indicated by the *gold spheres* (*bottom panel*). *B*, the data (mean ± S.D.) were quantified from four independent experiments with 5 to 6 images per experiment and expressed as the % of PAR1-LysoTracker contacts after SFLLRN stimulation and analyzed by one-way ANOVA followed by Tukey’s *post hoc* test, EV vs. WT, ∗∗∗∗, *p* < 0.0001, EV vs. YF, ∗∗∗∗, *p* < 0.0001, EV vs. YE, ns = not significant, WT vs. YF, ns = not significant, WT vs. YE, ∗∗∗∗, *p* < 0.0001, YF vs. YE, ∗∗∗∗, *p* < 0.0001. *C*, Cartoon of agonist peptide SFLLRN activated PAR1 induced internalization, association with ARRDC3 and lysosomal trafficking.

**Figure 9:**
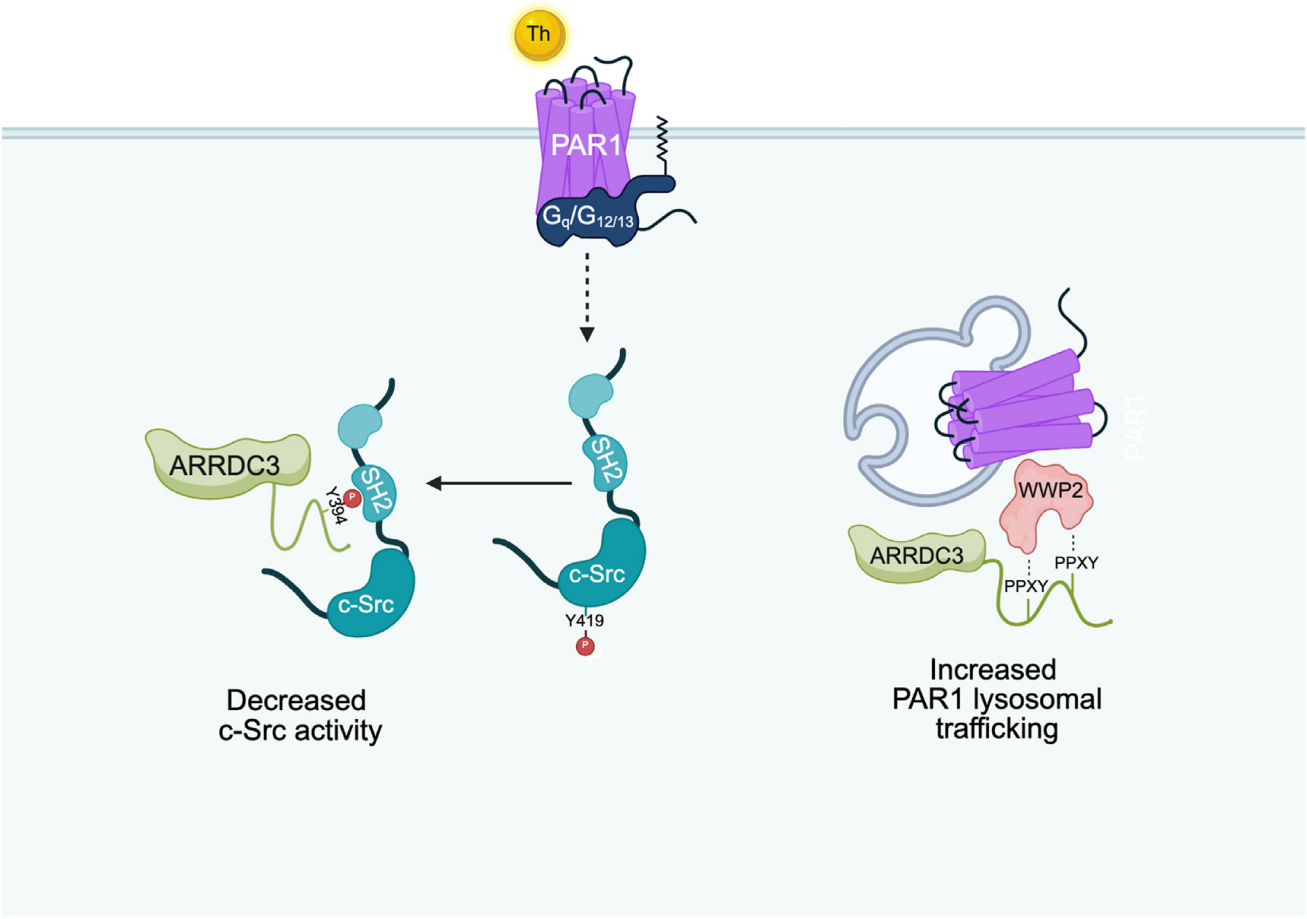
**ARRDC3 Y394 phosphorylation functions as regulatory switch for the PPxY motif**. Cartoon depicting phospho-regulation of ARRDC3 distinct scaffolding functions. In the absence of phosphorylation ARRDC3 PPxY motifs bind to the WW domains of WWP2 to facilitate thrombin-activated PAR1 lysosomal trafficking, whereas thrombin-induced ARRDC3 Y394 phosphorylation promotes binding to the SH2 domain of c-Src decreasing c-Src activity.

Agonist-activated PAR1 lysosomal trafficking requires ARRDC3-mediated recruitment of the WWP2 E3 ubiquitin ligase and ubiquitination of ALIX (19). To determine the functional impact of ARRDC3 Y394 phosphorylation on PAR1 trafficking, we used immunofluorescence confocal microscopy. HeLa-PAR1 ARRDC3 KO cells were transiently transfected with pEF.6 vector, ARRDC3 wild-type, Y394F, or Y394E, and stimulated with the PAR1 agonist peptide SFLLRN. Cells were then immunostained with antibodies to detect PAR1 and ARRDC3, labeled with LysoTracker Deep Red to image lysosomes, and quantified by surface rendering. In the absence of agonist stimulation, PAR1 localized to the cell surface in cells expressing ARRDC3 wild-type, Y394F and Y394E mutants, or pEF.6 vector (Fig. 8*A* top row panels). Whereas ARRDC3 wild-type, Y394F, and Y394E mutants resided primarily on intracellular vesicles in cells treated with or without agonist (Fig. 8, second and fourth row panels), indicating that the subcellular localization of ARRDC3 mutants remains intact. However, ARRDC3 Y394F and Y394E endosome surface volume was greater than wild-type ARRDC3 in cells incubated with the peptide agonist (Fig. S3*A*). After agonist stimulation, PAR1 was largely re-localized from the cell surface to lysosomal vesicles in control cells expressing pEF.6 vector only (Fig. 8*A* third row panels, and *B*). In cells co-expressing ARRDC3 wild-type or Y394F non-phosphorylatable mutant, lysosomal trafficking of activated and internalized PAR1 was significantly increased compared to pEF.6 vector only (Fig. 8, *A* and *B*). These results suggest that ARRDC3 Y394 phosphorylation is not required for PAR1 lysosomal sorting. However, in cells expressing the ARRDC3 Y394E phospho-mimetic, agonist-induced lysosomal sorting of PAR1 was significantly decreased compared to ARRDC3 wild-type and Y394F mutant (Fig. 8, *A* and *B*). Additionally, PAR1 lysosome surface volume was substantially less in cells expressing ARRDC3 Y394E ARRDC3, relative to EV, WT, and Y394F ARRDC3 (Fig. S3*B*). These findings suggest that perturbing the WWP2-ARRDC3 interaction *via* ARRDC3 Y394 phosphorylation or Y394E mutation functionally impacts sorting of PAR1 to the lysosome (Fig. 8*C*). Overall, this study demonstrates the importance of ARRDC3 Y394 phosphorylation for binding to selective partners, c-Src *versus* WWP2, and the functional impact on agonist-induced PAR1 signaling and lysosomal trafficking (Fig. 9).

## Discussion

In the present study we report the identification of a new α-arrestin ARRDC3 C-tail Y394 phospho-regulatory site that functions as a switch to enable distinct ARRDC3 binding partners and scaffolding functions. Previously we showed that ARRDC3 PPxY motifs bind to WW domains of the WWP2 E3 ubiquitin ligase (11). Here we report that phosphorylation of Y394 of the PPxY motif mediates interaction with c-Src *via* its SH2 domain. We also found that a non-phosphorylatable ARRDC3 Y394 mutant fails to bind to c-Src but retains binding to WWP2, whereas an ARRDC3 Y394 phospho-mimetic retained binding to c-Src and failed to associate with WWP2. Our results further show that ARRDC3 Y394 phosphorylation and c-Src SH2 domain-dependent interaction enables regulation of c-Src activity, whereas ARRDC3 Y394 phosphorylation disrupts WWP2 interaction and perturbs receptor trafficking. Together these findings indicate that ARRDC3 Y394 phosphorylation functions as a phospho-regulatory switch to enable selective binding to different partners that impact distinct scaffolding functions.

Several studies have shown that β-arrestin-1, -2, and visual arrestins interact with c-Src (8, 9, 33). Although ARRDC3 can dimerize with β-arrestins (23), β-arrestins do not mediate ARRDC3 interaction with c-Src, since we found that ARRDC3-c-Src interaction was retained in β-arrestin-1, -2 CRISPR/Cas9 gene-edited knockout cells. The mechanism of β-arrestin-c-Src interaction is most clear for β-arrestin-1. The N-domain of β-arrestin-1 contains three PxxP motifs that interact with the SH3 domain of c-Src (9). In addition, β-arrestin-1 also interacts with the SH1 kinase domain of c-Src (8). A new study indicates that the SH3 domain of c-Src binds tightly to activated β-arrestin-1 in an *in vitro* reconstituted system with purified proteins, whereas the SH1 domain prefers binding to the inactive form of β-arrestin-1 (34). In contrast to β-arrestin-1, visual arrestins lack proline motifs and interact with c-Src *via* the SH2 domain (33). We also demonstrate that ARRDC3-c-Src interaction occurs *via* the SH2 domain and a newly identified Y394 phosphorylated residue embedded in a semi-conserved YxEI/V c-Src SH2 domain binding consensus site (30). While several visual arrestin C-tail phosphorylated tyrosine residues have been reported (25), these Y phospho-sites are not part of a c-Src SH2 binding pYEEI consensus site, and their function in c-Src binding has not been reported. Thus, in contrast to visual arrestin, we defined the ARRDC3 Y394 phospho-site and c-Src SH2 domain as the determinants that specify interaction.

Unlike β-arrestins, the mechanisms that govern mammalian α-arrestin function are not well understood. The α-arrestin family includes 14 yeast members that are highly phosphorylated and 6 mammalian α-arrestin members with limited reports of phosphorylation (35). The functional implications of yeast α-arrestin phospho-regulation has been extensively documented (36, 37), whereas phospho-regulation of mammalian α-arrestins is emerging and best studied for TXNIP. Serine/threonine phosphorylation is dominant in yeast, and like yeast α-arrestins, TXNIP has been shown to be regulated by serine phosphorylation. A study showed that TXNIP phosphorylation at S308 promotes TXNIP mis-localization and degradation and thereby inhibits glucose transporter internalization and uptake (26, 38, 39). ARRDC3 tyrosine phosphorylation has also been reported, where ARRDC3 Y382 phosphorylation induced by insulin was shown to promote insulin receptor-ARRDC3 interaction and dampening of receptor activity (26). Surprisingly we found that the Y382 phospho-site is not required for ARRDC3 interaction with c-Src. Rather, our study identified a new Y394 phosphorylated site that is critical for c-Src SH2 domain-mediated interaction with ARRDC3.

The function of tyrosine phosphorylation as a regulatory switch for PPxY motifs was previously demonstrated for the β-dystroglycan protein (40). The β-dystroglycan protein PPxY motif binds to the WW domain of the dystrophin protein (41), whereas tyrosine phosphorylation promotes interaction with several SH2 domain-containing proteins including c-Src and Fyn (40). Studies have also reported that tyrosine phosphorylation of the PPxY motif in TXNIP acts as a regulatory switch that modulates binding to selective partners *in vitro*. These studies used purified WW domains of Itch E3 ligase and SH2 domains of SH2-containing protein tyrosine phosphatase-2 (SHP2) together with phosphorylated and non-phosphorylated PPxY peptides of TXNIP and showed that Y phosphorylation is required for SHP2 SH2 domain interaction and diminishes Itch WW domain binding (42). In endothelial cells, wild-type TXNIP was shown to bind to SHP2, whereas a Y378A mutant of the PPxY motif failed to associate with SHP2 (43). However, in that study, the phosphorylation status of the Y378 site was not determined. SHP2 is known to promote c-Src activation by regulating phosphorylation of the C-terminal Src kinase, which controls c-Src activity through Y527 phosphorylation (44). In the present study, we used mass spectrometry to identify Y394 phosphorylation and subsequently showed Y394 to function as a regulatory switch for the ARRDC3 PPxY motif. Indeed, we found that an ARRDC3 non-phosphorylatable Y394 mutant binds preferentially to WWP2, which occurs through WW domain interaction (11) whereas an ARRDC3 Y394 phospho-mimetic favors SH2 domain-mediated interaction with c-Src. Moreover, the loss of ARRDC3-c-Src interaction observed with the non-phosphorylatable Y394 mutant increases c-Src activity, suggesting that ARRDC3 suppresses c-Src activity. This is consistent with the ARRDC3 tumor suppressor function (21, 45, 46). Similarly, the loss of TXNIP binding to SHP2 SH2 domain increased c-Src activity (43), indicating that TXNIP suppresses c-Src activity through binding to the SH2 domain of SHP2.

In summary, our study demonstrates that the mammalian α-arrestin ARRDC3 is subjected to tyrosine phosphorylation, which functions as a regulatory switch for PPxY motifs. In addition to c-Src, ARRDC3 has been shown to associate with other Src family members, including Fyn and Lyn (22) but whether their interaction with ARRDC3 is governed similarly is not known. Thus, like β-arrestins, ARRDC3 displays multiple functions, and this study shows that phospho-regulation of ARRDC3 is linked to distinct scaffolding functions that facilitate interaction with different types of proteins to control distinct biological processes.

## Experimental procedures

### Reagents and antibodies

The following reagents were used in this study including human α-thrombin (#HT1002a, Enzyme Research Technologies), EGF (#AF-100–15, PreproTech), Protein A-Sepharose CL-4B beads (#GE17–0780–01, GE Healthcare), Protein A/G Magnetic Beads (#88846, Pierce), and LysoTracker Red DND-99 (#L7528, Thermo Fisher Scientific). Anti-PAR1 antibody C5433 was generated in our lab using the N-terminal PAR1 amino-terminal peptide sequence YEPFWEDEEKNESGLTEYC as described (47). Anti-HA conjugated to horseradish peroxidase (HRP) (#11667475001, Roche), anti-WWP2 (sc-166240, Santa Cruz Biotechnology), anti-Vinculin (#V9131, Sigma-Aldrich), anti-ARRDC3 (#ab64817, Abcam), anti-WWP2 (#ab103527), and HRP-conjugated goat anti-mouse (#170–6516) and goat anti-rabbit (#170–6515) were from BioRad. Anti-HA (#3742), anti-Myc (#2276), anti-β-arrestin1,2 (#4674), anti-phospho-Src family (Tyr416) (#2101), anti-c-Src (#2109), anti-Yes (#3201), anti-Fyn (#4023), anti-Lyn (#2732), anti-phospho-MYPT (#4563), anti-MYPT (#2634), anti-HA-tag (Alexa Fluor 488 Conjugate) (#2350), and rabbit IgG (#2729) antibodies were from Cell Signaling Technology. All commercially available antibodies are validated as described from the manufacturer websites.

### Cell culture and CRISPR/Cas9 gene editing

HeLa cells stably expressing FLAG-PAR1 (48) were maintained in DMEM supplemented with 10% fetal bovine serum (FBS) and 250 μg/ml hygromycin. HEK293T cells were purchased from ATCC and maintained in DMEM supplemented with 10% FBS.

HeLa-PAR1 CRISPR/Cas9 gene-edited ARRDC3 knockout cells were generated using the TrueTag Knockout enrichment kit (#A53815, Thermo Fisher Scientific), following the manufacturer’s instructions. The guide RNA (5′-CCACTCGCTACCTCATTCGA-3′) and homology arm primers for ARRDC3 (forward primer: 5′-CTTTATATTCTGCACAGACCACTCGCTACCTCATTCGGAAGTGGCTCAGGTTCTGGA-3′ and reverse primer: 5′-TTCACCCAATAGCGCACACTGCCATGTCGGCCTTCCTTGGCCGATCGCATACAGAG-3′) were designed using the Invitrogen TrueDesign Genome Editor. CRISPR/Cas9 gene-edited ARRDC3 KO HeLa-PAR1 cells were cultured at 37 °C with 5% CO2 in complete DMEM and selected in the presence of 10 μg/ml blasticidin and 2 ug/ml puromycin. The enriched gene-edited cells expressing both GFP and RFP were sorted by fluorescence-activated cell sorting and mass cell populations were routinely tested for *Mycoplasma* infection (#MP0025, Sigma-Aldrich).

### Cell transfections

Plasmid cDNA transfections were performed using 1 μg/ml polyethyleneimine (PEI) at a 3:1 ratio (3 μl PEI:1 μg plasmid). Wild-type HA-ARRDC3 cDNA cloned into the pEF6 vector was provided by Dr Quan Lu (Harvard University). HA-ARRDC3-Y382 F, HA-ARRDC3-Y394 F, HA-ARRDC3-Y394 E and HA-ARRDC3 truncation mutants were generated by site-directed mutagenesis (#12368010, Thermo Fisher). Myc-c-Src wild-type, SH3 domain W121A and SH2 domain R178A mutant cDNA plasmids were from Dr Steve Caplan (University of Nebraska).

HeLa-PAR1 cells were transfected using Oligofectamine 3000 (Life Technologies) and 50 nM siRNA per the manufacturer’s instructions. All single siRNAs were purchased from Qiagen: c-Src #7 5′-CGGCTTGTGGGTGATGTTTGA-3′ (#102223928) and #10 5′-CTCCATGTGCGTCCATATTTA-3′ (102,664,151) and AllStars negative control NS siRNA 5′-CUACGUCCAGGAGCGCACC-3′ (#1027281).

### Immunoprecipitations and immunoblotting

HEK293T cells were grown in 60 mm plates (6 × 10^5^ cells/plate) overnight at 37 °C and transfected with cDNA plasmids using PEI for 48 h. Cells were washed and lysed with NP-40 buffer (1% NP-40, 50 mM Tris- HCl, pH 7.4, 150 mM NaCl, 5% glycerol, 10 mM leupeptin, 1 ug/ml pepstatin, 10 mM aprotinin, 10 mM trypsin protease inhibitor, 10 mM benzamide, 1 mM PMSF, 5 mM NaF, 10 mM sodium pyrophosphate, and 1 mM Na_3_VO_4_). Lysates were homogenized and clarified by centrifugation at 14,000 rpm for 30 min at 4 °C. Clarified lysates were quantified by bicinchoninic acid assay (Thermo Fisher Scientific) and incubated with the indicated antibodies and Protein A Sepharose beads or anti-HA magnetic beads overnight at 4 °C. Protein-bound beads were washed, resuspended in 2X Laemmli sample buffer (LSB) with DTT, resolved by SDS–PAGE gel, transferred to PVDF membrane and immunoblotted as indicated. Immunoblots were quantified by densitometry using ImageJ software (NIH). Quantification of protein expression was determined by normalizing the integrated intensity signal to the average signal of each replicate and expressed as a fraction relative to the average of the indicated control group.

### Mass spectrometry

HEK293T cells (1.5 × 10^6^ cells per 10-cm dish) were transiently transfected with HA-ARRDC3 P401R variant using PEI. Cells were serum starved and stimulated with EGF (50 ng/ml) or thrombin (10 nM) for 5 min at 37 °C. Cells in two 10-cm dishes were used per condition and lysed with NP-40 buffer (50 mM Tris-HCl, pH7.5, 150 mM NaCl, 1 mM EDTA, 1% NP-40, 5% glycerol, 1 ug/ml pepstatin, 10 mM leupeptin, 10 mM aprotinin, 1 mM PMSF, 10 mM TPI, 10 mM benzamide, 1 mM NaVO_4_, 5 mM NaF, 10 mM NaPP, plus one tablet PhosSTOP (#04906837001, Roche) per 10 ml buffer. Lysates were homogenized, solubilized at 4 °C, and centrifuged at 14,000 rpm for 20 min at 4°C. HA-ARRDC3 was purified from supernatant using anti-HA magnetic beads (#88836, Pierce) at 4 °C, washed 3X with wash buffer (same as lysis buffer, without glycerol), followed by ultrapure H_2_O. HA-ARRDC3 protein was eluted in 1x LSB (no DTT) and heated for 10 min at 95 °C. DTT was added, before loading the entire eluate on a 7% SDS-PAGE gel. After electrophoresis the gel was stained in 0.1% Coomassie Blue in 10% acetic acid/50% methanol for 30 min and destained in 10% acetic acid/50% methanol until bands were visible. The prominent band corresponding to the molecular size of ARRDC3 was excised and submitted for MS analysis.

In gel digest was conducted as described (49). Briefly, gel slices were destained, processed, digested with ice-cold trypsin (0.01 μg/ml), and peptides extracted. Trypsin-digested peptides were analyzed by ultra-high pressure liquid chromatography (UPLC) coupled with tandem mass spectroscopy (LC-MS/MS) using nano-spray ionization using a TimsTOF 2 pro hybrid mass spectrometer (Bruker) interfaced with nanoscale reversed-phase UPLC (EVOSEP ONE). Evosep method of 30 SPD (samples per day) was utilized using a 10 cm × 150 μm reverse-phase column packed with 1.5 μm C18-beads (PepSep, Bruker) at 58 °C. The analytical columns related to a fused silica ID emitter (10 μm ID; Bruker Daltonics) inside a nanoelectrospray ion source (Captive spray source; Bruker). The mobile phases comprised 0.1% FA as solution A and 0.1% FA/99.9% ACN as solution B. The mass spectrometry setting for the TimsTOF Pro 2 are as following: PASEF method for standard proteomics. The values for mobility-dependent collision energy ramping were set to 95 eV at an inversed reduced mobility (1/*k*_0_) of 1.6 V s/cm^2^ and 23 eV at 0.73 V s/cm^2^. Collision energies were linearly interpolated between these two 1/*k*_0_ values and kept constant above or below. No merging of TIMS scans was performed. Target intensity per individual PASEF precursor was set to 20,000. The scan range was set between 0.6 and 1.6 V s/cm^2^ with a ramp time of 166 ms. 14 PASEF MS/MS scans were triggered per cycle (2.57 s) with a maximum of seven precursors per mobilogram. Precursor ions in an *m*/*z* range between 100 and 1700 with charge states ≥3+ and ≤8+ were selected for fragmentation. Active exclusion was enabled for 0.4 min (mass width 0.015 Th, 1/*k*_0_ width 0.015 V s/cm^2^). Protein identification and label free quantification was carried out using Peaks Studio X (Bioinformatics solutions Inc.).

### Molecular modeling

The structural model of a complex between c-Src SH2 (aa 144–249) and a C-terminal phosphopeptide from ARRDC3 (aa 390–402 with pY394) was built by homology in ICM 3.9-3b (Molsoft LLC) using the structure complex of c-Src SH2 in complex with a phosphopeptide EPQ-pTyr-EEI (PDB 7T1U) (50) as a template. The model was refined by tethering the backbone atoms of the peptide to the corresponding atoms in the template and by running 10^5^ steps of biased probability Monte Carlo optimization (51) the peptide and the side-chains of c-Src interface residues, both represented as full-atom models in internal coordinates. The structural model of a complex between the C-terminal peptide of ARRDC3 (residues 390–402 with no pTyr) and WWP2 WW2 (aa 296–369) was built using AlphaFold2 on Multimer v2.3.2 (52) locally installed on the UCSD Triton Shared Computing Cluster (TSCC) and refined similarly.

### Signaling assays

Signaling assays were performed as described (21). Briefly, HeLa-PAR1 ARRDC3 KO cells were transfected, starved overnight and stimulated with 10 nM thrombin for the indicated times at 37 °C. Cells were lysed in Triton X-100 buffer (50 mM Tris-HCl, pH 7.4, 100 mM NaCl, 5 mM EDTA, 50 mM NaF, 10 mM sodium pyrophosphate, and 1% Triton X-100), quantified, and diluted in 2x LSB with 200 mM DTT. Equivalent amounts of cell lysates were resolved by SDS-PAGE and immunoblotted as indicated.

### Immunofluorescence confocal microscopy

HeLa-PAR1 CRISPR/Cas9 ARRDC3 KO cells were plated on fibronectin-coated glass coverslips in 6-well plates (1 × 10^5^ cells/well), grown overnight and transfected with HA-ARRDC3 and pEF.6 vector cDNA plasmids as indicated for 48 h. Cells were washed, serum-starved for 1 h, incubated with LysoTracker, and stimulated with 100 μM SFLLRN agonist peptide for indicated times at 37 °C. Cells were fixed in 4% paraformaldehyde, washed, and incubated with rabbit anti-PAR1 antibodies and anti-HA mouse antibody -conjugated to AlexaFluor-488 diluted in 1x PBS containing 0.5% bovine serum albumin and 0.2% saponin. Coverslips were mounted with ProLong Gold reagent (Thermo Fisher). Confocal images XY sections were collected sequentially using an Olympus IX81 DSU spinning confocal microscope fitted with a Plan Apo 60X 1.4 NA oil objective and a Hamamatsu ORCA-ER digital camera using Metamorph version 7.7.4.0 software (Molecular Devices).

### Imaris image analysis

Z-sections of PAR1 and Lysotracker images (7 slices) were 3D-rendered with Imaris x 64 10.1 software (Bitplane AG) coupled with custom MATLAB (2020 and 2023) programming, as described (53). Briefly, for Imaris analysis, the image display was adjusted for the channels, and rendering quality was set to 100%; 3D surfaces were generated by selecting the source channel with the following creation parameters. PAR1 and Lysotracker endosomal surface grain size were 0.150 μm and 0.115 μm, diameter of the largest sphere was 0.700 μm and 0.606 μm, manual threshold values were 25.4605 μm and 3.07148 μm and region growing estimated diameter were 0.600 μm and 0.500 μm and quality above were 7.26 μm and 1.5 μm, respectively. ARRDC3 surface grain size was 0.250 μm, diameter of the largest sphere was 0.250 μm, region growing estimated diameter was 0.300 μm, and quality was above automatic threshold. To quantify the surface contacts (colocalization or overlap) between 3D-rendered surfaces, the Imaris XT bundle “Kiss and Run” was launched and the rendered images were then processed using the Distance Transformation module with a threshold for the distance between surfaces set at 0 μm, where 3D surfaces touching each other are considered contacts (overlap). Percentage surface contacts were then quantified and exported to Excel for further analysis. Percentage surface contacts were calculated using Imaris XT bundle “Kiss and Run” distance transform module with Lysotracker set as target surface and PAR1 as the tracked surface.

### RT-qPCR

RNA was isolated from HeLa-PAR1 parental and ARRDC3 KO cells seeded at 1 × 10^5^ cells per well of a 24-well plate and RNA was extracted using Direct-zol RNA Miniprep Plus Kit (#R2072, Zymo Research). Total RNA was quantified and 1 μg total RNA was utilized for cDNA synthesis reaction with the SuperScript IV VILO Master Mix with ezDNase enzyme kit (#111766050, Thermo Fisher Scientific). Quantitative RT-PCR was performed with TaqMan Fast Advanced Master Mix (#4444964, Thermo Fisher Scientific) and TaqMan Gene Expression Probes ARRDC3 (#Hs01055314_m1), using a QuantStudio 3 Real-Time PCR System (Thermo Fisher Scientific). ARRDC3 mRNA transcript levels were analyzed using the comparative CT (threshold cycle) method, normalized to 18S ribosomal RNA expression. Briefly, the number of cycles until threshold (Ct) was determined for each target, and the Ct value for 18S was subtracted from the Ct value for each target, the differences in expression was then determined using the 2^-ΔΔCt^ method.

### Statistical analysis and software

Data were analyzed using Prism 10.4.1 (GraphPad Software) and Microsoft Excel. Statistical analysis methods are indicated in the figure legends. Figures were created in Adobe Illustrator and Photoshop and cartoons were created with BioRender. PAR1-lysotracker overlap was determined using Imaris Software and Jalview was used to align arrestin sequences.

## Data availability

The data are contained within the article or shown as supplemental data. The mass spectrometry proteomics data have been deposited to the ProteomeXchange Consortium *via* the PRIDE [1] partner repository with the dataset identifier PXD060782.

## Supporting information

This article contains supporting information.

## Conflict of interest

The authors declare that they have no conflicts of interest with the contents of this article.
