# Supplementary material for: ARRDC3 tyrosine phosphorylation functions as a switch to control c-Src versus WWP2 interactions and distinct scaffolding functions
Source: J Biol Chem. 2025 May 21;301(7):110270. doi: 10.1016/j.jbc.2025.110270 (PMC12213294; doi:10.1016/j.jbc.2025.110270)
Supplement: Supporting Information [file mmc1.pdf]

Supporting Information

Title:

ARRDC3 tyrosine phosphorylation functions as a switch to control c-Src versus WWP2 interactions and distinct scaffolding functions

Authors:

Mika Caplan, Carolyne Bardeleben, Kanika Dhawan, Rhea Plawat, Irina Kufareva, and JoAnn Trejo

List of supplemental materials

Table S1. ScanSite 4.0 predicted motif sites for ARRDC3

Fig. S1. Schematic of ARRDC3 motif sites that are likely to be phosphorylated by specific kinases.

Fig. S2. ARRDC3 phospho-proteomic analysis

Fig. S3. ARRDC3 and PAR1 puncta surface volume

Table S1. ScanSite 4.0 predicted ARRDC3 motif sites that are likely to be phosphorylated by specific kinases.

motif gene symbol	motif name	motif group	score	percentile	site	site sequence
PRKCE	PKC epsilon	Basophilic serine/threonine kinase group	0.3593	0.0010111	T157	VKLKKEF**t**VFEHIDI
AKT1	Akt Kinase	Basophilic serine/ threonine kinase group	0.4391	0.00123927	S323	PFGSRTS**s**VSSQCSM
FYN	Fyn SH2	Src homology 2 group	0.3114	4.45E-04	Y394	RFLPPPL**y**SEIDPNP

Fig S1

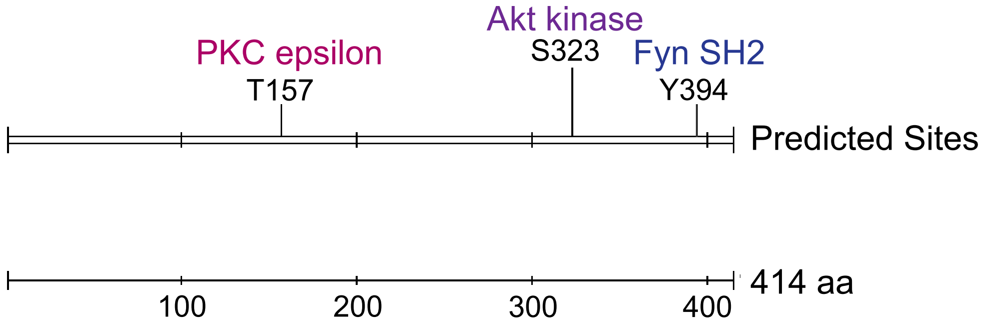

Fig. S1. Schematic of ARRDC3 motif sites that are likely to be phosphorylated by specific kinases. ScanSite 4.0 software predicted ARRDC3 Y394 to bind to SH2 domain of Fyn, a member of the Src family of kinases. Other kinases predicted to bind to and phosphorylate ARRDC3 include protein kinase C epsilon (PKC-ε) and Akt.

Fig S2

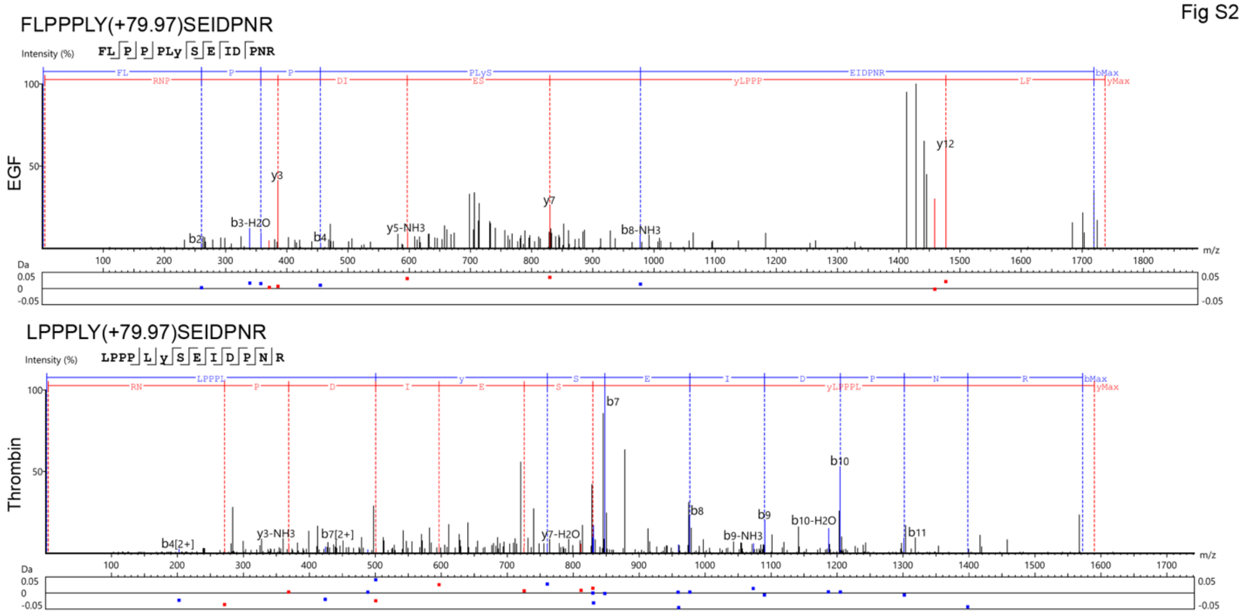

Fig. S2. Phospho-proteomics spectra of ARRDC3 Y394 phosphorylation. Spectra of peptides detected in epidermal growth factor (EGF) and thrombin stimulated HEK293 cells expressing HA-ARRDC3.

Fig S3

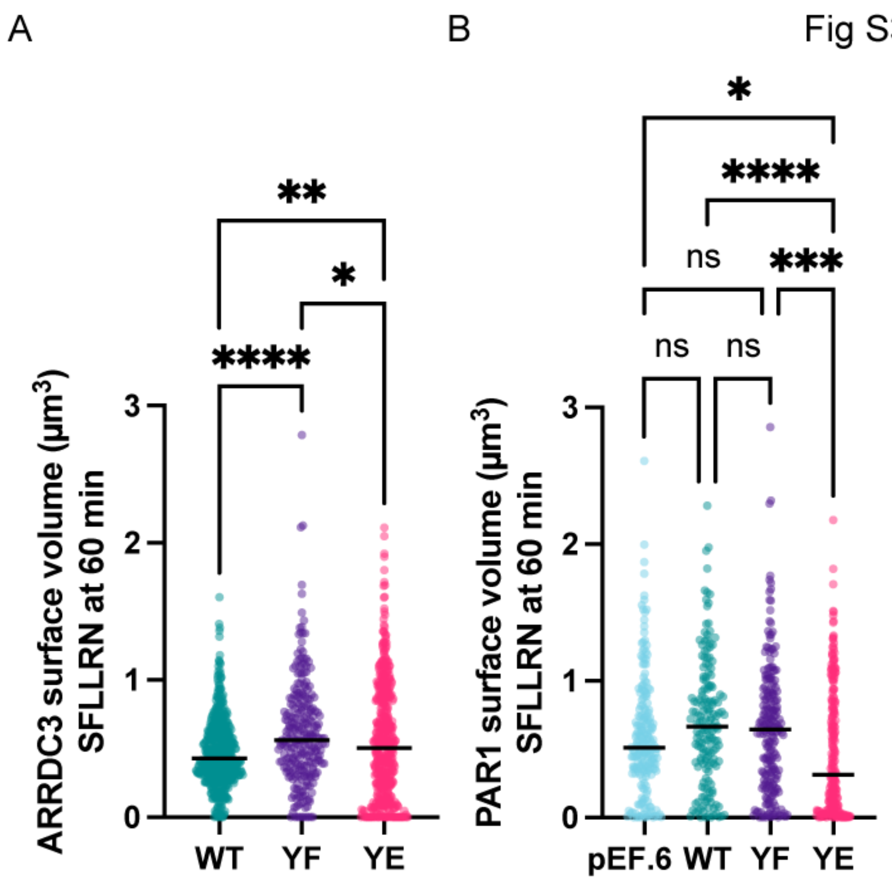

Figure S3. ARRDC3 mutants affect ARRDC3 and PAR1 puncta sizes. *A-B,* HeLa-PAR1 ARRDC3 CRISPR/Cas9 KO cells expressing HA-ARRDC3 (WT), Y394F, Y394E, or pEF.6 empty vector were stimulated with agonist peptide SFLLRN, processed and immunostained for HA-ARRDC3 and PAR1. Puncta sizes were quantified using Imaris software. The data were quantified using 8 to 10 images per condition and analyzed by one-way ANOVA followed by Tukey's post hoc test, ARRDC3 surface volume, WT vs. YF, ****, $p < 0.0001$, WT vs. YE, **, $p = 0.0027$, YF vs. YE, *, $p = 0.0457$. PAR1 surface volume, pEF.6 vs. YE, *, $p = 0.0483$, WT vs. YE, ****, $p<0.0001$, YF vs. YE, ***, $p = 0.0006$, ns = not significant.
